# Australian Troops' Bravery

**Published:** 1918-07-27

**Authors:** 


### THE EMPIRE AROUSED.

### Australian Troops' Bravery.

The Germans continue their foul work without intermission. In the *Times* of the 20th inst. the Admiralty announces that H.M. Transport *Barunga* (ex German s.s. *Sumatra*), outward bound to Australia, conveying unfit Australian troops, was torpedoed and sunk by a German submarine on July 15. On page 359 will be found a statement of the decision and feeling of Canadians upon these outrages, and the effect they must have, and indeed have had, throughout Canada and throughout the Empire. With regard to this greatest and worst of her crimes the united feeling is that there can be no peace until Germany has been brought to her knees. Germany, Sir Edward Kemp declared, has made a wall over which she will not be able to trade with Canadians, and let us hope with the united nations embraced by the alliance, for generations to come.

### A Lesson in Discipline.

By the courtesy of the United Cable Service (Australasia) the *Times* was enabled to publish the following account of the wreck of the *Barunga*, a steamer of 7,484 tons, built at Flensburg in 1913:—

Magnificent Australian discipline marked the sinking of the *Barunga* on Monday, July 15. The ship was crowded with invalids returning to Australia when it was torpedoed in the forward hold. The first and second bulkheads collapsed, but happily the engine-room bulkhead held, so that the ship floated for a considerable time, enabling all to reach safety on board the destroyers which came to the rescue. The explosion, which was terrific, shook the *Barunga* from stem to stern, hurling everyone to the deck. The gun crew were flattened out, but quickly jumped to their feet again and fired a shot at a range of 300 yards, striking the water just beyond the submarine, which dived. In the hospital, forward, nurses were thrown to the floor, patients tossed from their berths, and medicine bottles smashed. The nurses behaved splendidly; all lights were extinguished, and the patients were helped to the decks.

### At the Boat Stations.

Despite the dreadful possibilities—for the ship appeared to be rapidly sinking, there was not the slightest panic. Fortunately, after settling for 10 ft., the boat remained stationary for about half an hour. The men lined up at the boat stations, helping those who were incapacitated, and at the same time singing Australian songs. Many on board had been fighting since the beginning of the war, and they included a dozen helpless cripples.

The steamer soon settled down by the head, and there was no time to save anything. Everyone was ordered to leave, and many men had to jump overboard in order to reach the rafts. British warships steamed up at full speed, and in some cases men were able to jump clear aboard. But for more than a hour the sea was an extraordinary spectacle. Boats, rafts, planks floated about in indescribable confusion, stretching far behind the ship as she plunged forward and then settled into a quiet dying movement. A large number of bales dribbled from the hole in the steamer's side. Men clung to all sorts of objects. Two of the destroyers got to windward so that the floaters should drift in their direction, while a third warship circled about, created an inward wash, and thus shepherding the floaters. Sometimes the scene resembled an aquatic carnival, and there was a constant flow of badinage between groups of swimmers and floaters. Some men paddled around, using hats as oars, while one, sitting safely alone on a raft, got a long stick, to which he tied a white handkerchief, and caused much amusement by frantic wavings for help.

### Finishing the Rubber.

On board the *Barunga* the time had been passed with typical nonchalance. Four soldiers were playing bridge on the mess-deck at the time the vessel was hit. Seeing that everything was quiet, they returned and finished their rubber before being ordered to take to the boats. A popular game in the early days on board ship was called "House." The banker had accumulated some thousands of pennies when the torpedo struck, so he brought these pennies and scattered them on the deck, saying, "Come on, diggers, help yourselves; these are too heavy for me to get away with." Soldiers say that the pennies were all salved.

### A Doctor's Bravery.

The officers pay a special tribute to Adjutant-Major Montgomery, A.A.M.C., for his action after the *Barunga* was struck. When nearly all had left the ship he discovered four soldiers leaning over the rails at the stern, and asked why they had not gone overboard. They said they could not swim, so handing each a lifebelt the adjutant said, "Well, you'll jolly well have to learn," and jumped in himself and saw them all safely on to rafts, afterwards swimming alongside the ship to see whether anyone had been left. All the soldiers and crew were clear of the ship within half an hour. The last to leave the *Barunga* were the ship's captain, Captain Wilson, and Colonel Burnage, the officer commanding the troops, and who was in the Dardanelles campaign.

### The Work of Rescue.

Throughout the work of rescue, first attention was given to those who were incapacitated, some shell-shock cases suffering terribly. Many who had been floating were completely exhausted when rescued, and it was a trying time resuscitating them on board the destroyers, as the water was extremely cold.

There was intense relief when the seas were cleared and everyone was in safety aboard. The *Barunga* sunk with a final shiver, and then the rescuing ships turned their bows homewards, and were soon landing the men—kitless and in all kinds of attire, but cheerful. The naval officers showed the utmost kindness, offering all their stocks of food and drink, and seeing that the bad cases were comfortably placed in the engine-room and forecastle for special attention. Being unaccustomed to the motion of a destroyer, many men had to hang on to the ropes and railings during the long hours before reaching land. Eight soldiers were sent to the hospital when put ashore, and the others have begun already to collect kit in preparation for another start. Two chaplains on board the destroyers were sea-sick, but were very helpful at the disembarkation. The officers threw off their tunics and boots after the explosion, and estimate that this loss of kit averages about £100 sterling. A number of them, clothed in variegated uniforms, unshaven, weary, but undaunted, arrived in London.

### The Nurses Bravely Alert.

The nurses received whole-hearted tribute. Lieutenant F. C. Symonds, of Melbourne, says that there were four nurses on board and they were "just lovely: their only concern was the welfare of the sick. Matron Blundell set a splendid example, though severely shaken by the explosion." Three thousand pounds worth of Red Cross goods for the men's comfort sank with the ship. There were nearly 900 soldiers on the boat, and the way in which all were rescued was a miracle of discipline, coolness, and organisation. "It was heart-stirring," said an officer, "to see such evidences of the men's courage, good-humour, and tenderness towards their suffering mates. What would one not do for such!" A New South Wales soldier summed up the situation by affirming: "It was a damned bad shot of Fritz's; if he'd hit us amidships we would have been neither here nor going to Australia."

